# Secondary Metabolites Produced by an Endophytic Fungus *Pestalotiopsis microspora*

**DOI:** 10.1007/s13659-019-00225-0

**Published:** 2019-11-15

**Authors:** G. R. Nalin Rathnayake, N. Savitri Kumar, Lalith Jayasinghe, Hiroshi Araya, Yoshinori Fujimoto

**Affiliations:** 1 National Institute of Fundamental Studies, Hantana Road, Kandy, Sri Lanka; 2 School of Agriculture, Meiji University, Kawasaki, Kanagawa 214-8571 Japan

**Keywords:** *Pestalotiopsis microspora*, *Manilkara zapota*, Endophyte, Pitholide E, Pestalotin

## Abstract

**Abstract:**

An endophytic fungus *Pestalotiopsis microspora* isolated from the fruits of *Manilkara zapota* was cultured in potato dextrose broth media. Chromatographic separation of the EtOAc extract of the broth and mycelium led to the isolation of a new azaphilonoid named pitholide E (**1**), in addition to previously identified pitholide B (**2**), pitholide D (**3**), pestalotin (LL-P880α) (**4**), PC-2 (**5**), LL-P880β (**6**), tyrosol (**7**) and 4-oxo-4*H*-pyran-3-acetic acid (**8**). An endophytic fungus *P. microspora* from *M. zapota* and the isolation of compounds **1**–**5**, **7** and **8** from *P. microspora* are reported here for the first time.

**Graphic Abstract:**

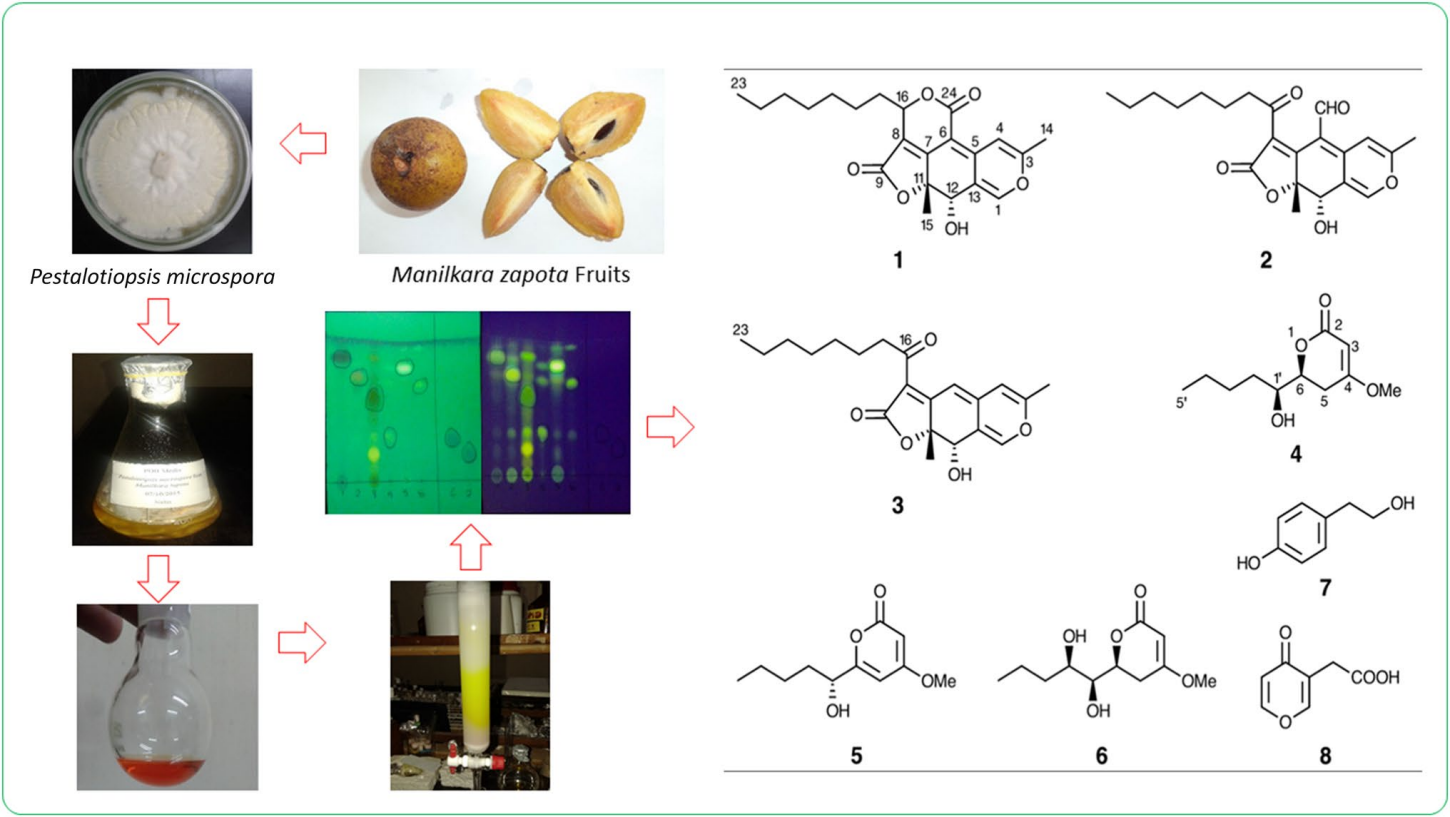

## Introduction

**Fig. 1:**
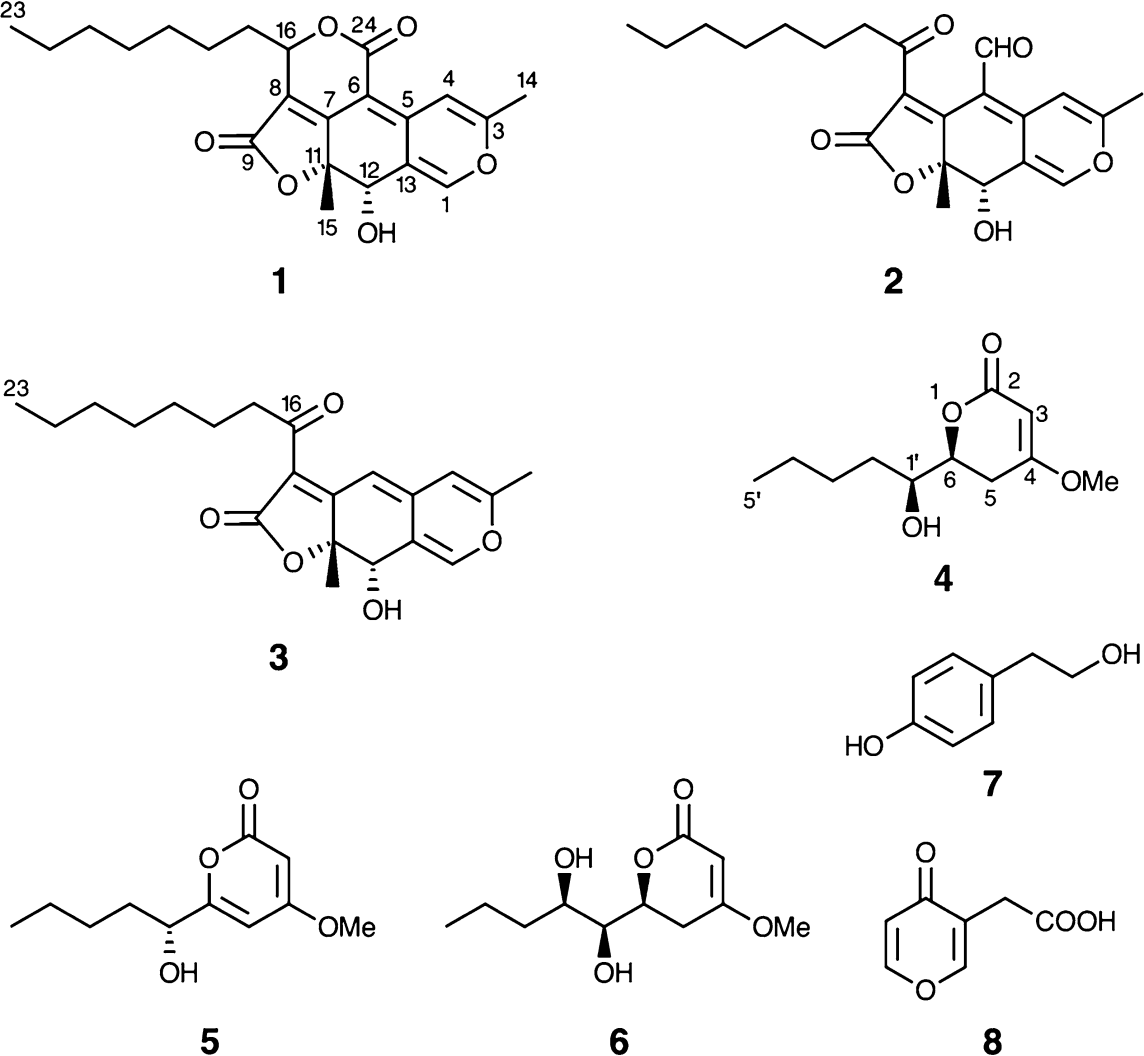
Structures of compounds **1**–**8**

Fungi are considered to be an important source of bioactive compounds. Fungi can be categorized into two groups, epiphytic fungi and endophytic fungi. Epiphytic fungi live on the surface of a host while endophytic fungi colonize inner tissues and even live in the cells of their hosts [1]. Some endophytic fungi have developed the ability to produce the same or similar bioactive substances as those originating from the host plants [2]. We have previously reported several bioactive compounds produced by endophytes isolated from Sri Lankan plants [3–10]. Recently we investigated the secondary metabolites produced by an endophytic fungus *Pestalotiopsis microspora*, isolated from the fruits of *Manilkara zapota* (local name: Sapodilla) of the family Sapotaceae. The fruits are popular in tropical countries and the leaves are used for antidiabetic treatment. Various antidiabetic and antilipidemic effects of *M. zapota* fruits have been reported [11]. In addition, the traditional uses, phytochemistry and pharmacological activity of the plant have been reviewed [12]. In this paper we report the isolation and identification of a new azaphilonoid named pitholide E (**1**) from a culture of *P. microspora*, in addition to already known pitholide B (**2**), pitholide D (**3**), pestalotin (LL-P880α) (**4**), PC-2 (**5**), LL-P880β (**6**), tyrosol (**7**) and 4-oxo-4*H*-pyran-3-acetic acid (**8**) (Fig. 1).

## Results and Discussion

An endophytic fungus isolated from the fruits of *M. zapota* was identified as *P. microspora* by morphological studies (cotton like mycelium grown with irregular margins, uniform conidia consists with five cells with three brown to fuliginous median cells and hyaline end cells) as well as by sequence analysis of the ITS region of the rDNA gene. Amplification of the ITS region was carried out using the universal eukaryotic primers of ITS1 and ITS4. BLAST search indicated that the sequence of the ITS region had 100% similarity to that of *P. microspora* JD1 (GenBank Accession No. KP231875.1). A pure culture of *P. microspora* (IFS/N/MZ/1/2014) and photographic evidence of the fruits of *M. zapota* and the strain are deposited at the National Institute of Fundamental Studies.

Silica gel TLC autobiography analysis [13] found that EtOAc extract obtained from the culture broth and from mycelium of *P. microspora* grown in potato dextrose broth (PDB) displayed antifungal activity against *C. cladosporioides*. In addition, antioxidant activity was also demonstrated by DPPH radical scavenging assay (IC_50_ 63.5 μg/mL) [14]. However, it was inactive in the assays of brine shrimp lethality [15], phytotoxicity against *Lactuca sativa* (inhibition of root and shoot growth) [16], α-amylase inhibition [17] and anticandidal activity against *Candida tropicalis* [18]. Chromatographic separation of the EtOAc extract over silica gel and Sephadex LH-20, and by preparative silica gel TLC furnished a new azaphilonoid pitholide E (**1**), in addition to previously identified pitholide B (**2**), pitholide D (**3**) [19], pestalotin (**4**) [20, 21], PC-2 (**5**) [22, 23], LL-P880β (**6)** [24, 25], tyrosol (**7**) [26] and 4-oxo-4*H*-pyran-3-acetic acid (**8**) [27, 28]. The structures of the known compounds were determined by comparing their spectroscopic data with literature values. TLC analysis indicated that none of these compounds were present in the MeOH extract of *M. zapota* fruits.

**Fig. 2:**
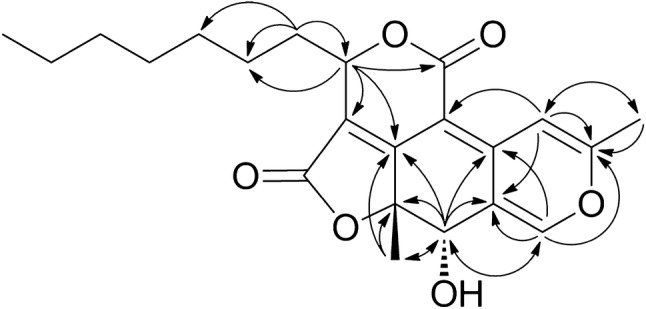
Key HMBC correlations for compound **1**

Compound **1** was isolated as luminous yellow oil. HRFABMS data indicated that it has a molecular formula of C_22_H_26_O_6_, with 10 degrees of unsaturation. The ^1^H and ^13^C NMR spectra, showing 22 carbon signals, resembled those of an azaphilonoid such as pitholide B (**2**) [19]. Compound **1** displayed three methyl signals at *δ* 2.29 (s), 1.44 (s) and 0.87 (t, *J* = 7.0 Hz) assignable to H_3_-14, H_3_-15 and H_3_-23, respectively, in the ^1^H NMR spectrum. In addition, the presence of an oxymethine proton at *δ* 4.53 (s, H-12) and two significantly deshielded olefinic protons at *δ* 7.54 (s, H-1) and 7.61 (s, H-4), supported a C_22_-pitholide structure. Detailed analysis of the NMR spectra in comparison with those of compound **2** suggested that the C-24 aldehyde in **2** was oxidized into an acid (appearance of *δ*_C_ 162.6 in **1** with disappearance of *δ*_H_ 9.71/*δ*_C_ 186.8 in **2**), while the C-16 ketone in **2** was reduced to an alcohol (appearance of *δ*_H_ 5.46 (dd, *J* = 7.0, 3.5 Hz)/*δ*_C_ 79.1 in **1** with disappearance of *δ*_C_ 198.6 in **2**), and these were cyclized to form a *δ*-lactone. The lactonization was supported by an HMBC correlation from H-16 to C-24 and a downfield shifted H-16 (*δ* 5.46) compared to that of an allylic secondary alcohol. Key HMBC correlations for compound **1** (Fig. 2) were consistent with the proposed tetra-cyclic structure. Complete ^1^H and ^13^C NMR assignments of compound **1**, attained with assistance of the HMQC spectrum, are described below.

**Fig. 3:**
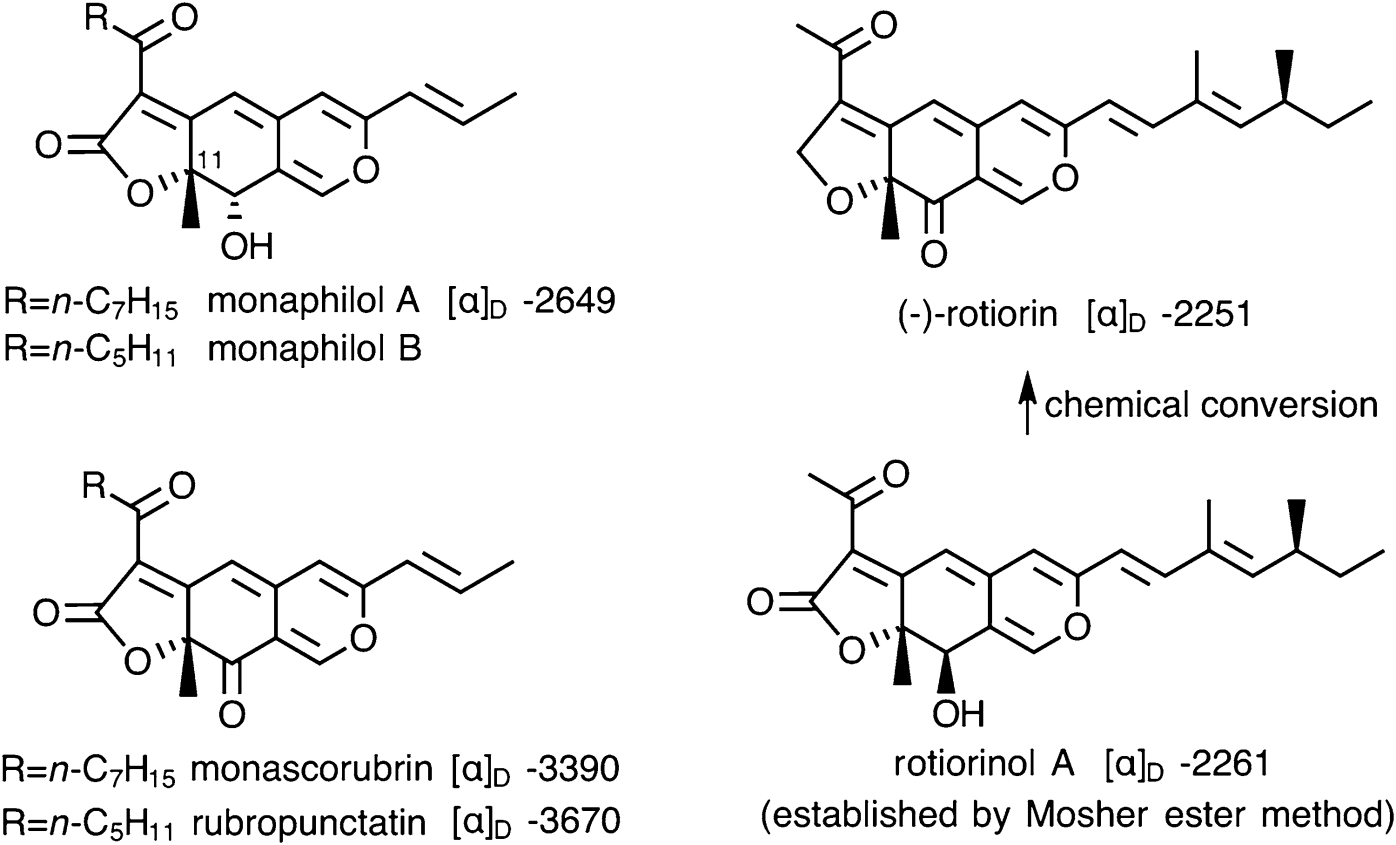
Correlation between C-11 configuration and a large negative specific rotation

Compound **1** is stereochemically homogenous at the C-16 position as evidenced by a single set of ^13^C NMR resonances. NOE correlation between H_3_-15 and H-16 was not observed in the NOE experiments (ROESY and differential NOE spectra irradiating H_3_-15 and H-16), although an intense NOE correlation was observed between H_3_-15 and H-12. These findings may suggest an *anti* relationship between H_3_-15 and H-16 (the proximity of H-16 and the nearest proton of H_3_-15 was calculated as 4.3 Å for the *syn*-isomer). Regarding the absolute stereochemistries at the C-11 and C-12 positions of pitholide E, 11*R*,12*S* configuration was proposed for monaphilols A and B, which have the same partial structure (*trans*-orientation of 11-Me and 12-hydroxy groups) as pitholides B, D and E, in view of a large negative sign of specific rotation [29]. The assignment is based on the assumption that the absolute configuration at the C-11 chiral center contributes the negative specific rotation. The assumption will be acceptable since structurally related 11*R* azaphilonoids (*cis*-orientation of 11-methyl and 12-hydroxy groups) such as rotiorinol A as well as 11*R* azaphilonoids with a 12-oxo group such as monascorubrin, rubropunctatin and (−)-rotiorin display a large negative specific rotation commonly (Fig. 3). The 11*R* configuration of rotiorinol A and (–)-rotiorin have been established unambiguously [30]. Hence, we propose 11*R*,12*S* configuration for pitholide B, D as well as pitholide E, although the stereochemical assignments should be ascertained by an unambiguous manner such as X-ray and ECD study. Our attempt to determine the absolute configuration of pitholide D using Mosher ester method was not successful since irregularities in the Δ(*δS* − *δR*) values were observed. Finally, our specimens of pitholides B and D showed much larger negative specific rotations ([α]_D_^25^ − 741 (*c*, 0.23, MeOH) for **2** and − 980 (*c*, 0.077, MeOH) for **3** than the reported values ([α]_D_ − 158 (*c*, 0.85, MeOH) for **2** and [α]_D_ − 248 (*c*, 0.51, MeOH) for **3** [19]).

*Pestalotiopsis microspora* is known as a taxol-producing fungus [31] and reported to have an ability to break down and digest polyurethane [32]. Pitholides B (**2**) and D (**3**) have been previously isolated, along with pitholides A and C, from *Pithomyces* sp*.* derived from a marine tunicate [19], and they structurally belong to an azaphilone class of polyketides. These pitholides are assumed to be biologically benign, for example, inactive in brine shrimp lethality assay [19]. (−)-Pestalotin (6*S*,1′*S*-form) (**4**) was first isolated from *Pestalotia cryptomeriaecola* [33] and has since been identified in several fungi including *Pestalotiopsis* sp. such as *P. karstenii* [34]. Epipestalotin (either 6*R*,1′*S-* or 6*S*,1′*R*-form) was reported from an endophytic fungus isolated from a tropical lichen *Everniastrum nepalense* [35]. (+)-(1′*R*)-PC-2 (**5**) which is also called dehydropestalotin has been reported from some endophytic fungi including *P. mangiferae* [36] and *Penicillium* sp.[22]. Specific rotation of **5** ([α]_D_^25^ + 76.5 (*c*, 0.62, MeOH)) was found to be identical to the literature value ([α]_D_ + 78.5 (*c*, 0.19, MeOH)) [22], implying it had a 1′*R* configuration. It is noteworthy that the C-1′ configuration of **5** is opposite to those of compounds **4** and **6**, even though compounds **4**–**6** are produced by the same fungus strain. The antipode of **5 (**1′*S-*form) ([α]_D_ − 77.1 (*c*, 0.52, MeOH) has also been identified in a few other fungi including *P. phitiniae* [37] and *Urnula craterium* [38]. (−)-LL-P880β (6*S*,1′*S*,2′*R*-form) (**6**) has been previously reported from some fungi including *P. microspora* isolated from *Taxus chinensis* [39]. The C-2′ epimer of **6** (6*S*,1′*S*,2′*S*-form) has been also reported from an endophytic fungus *P. virgatula* obtained from mangroves [40]. Compound **4** was reported to act as a plant growth regulator, as a gibberellin synergist [33], while compounds **4** and **6** have moderate cytotoxicity towards several cancer cell lines [34, 41]. Tyrosol (**7**) is a commonly occurring fungal metabolite and has been isolated from endophytic fungi *Pestalotiopsis* sp. including *P. versicolor* [42] and *P. virgatula* [43]. The tyrosine metabolite is widespread in *Candida* species and reported as a quorum-sensing molecule in the yeast *C. albicans* [44]. 4-Oxo-4*H*-pyran-3-acetic acid (**8**) has been isolated from several fungi including *Pestalotiopsis* sp [45]. Compounds **1**, **4, 6** and **7** were evaluated for antioxidant and antifungal activity against *C. cladosporioides*, but none of them showed any significant activity. In conclusion, the endophytic fungus *P. microspora* was isolated from *M. zapota* for the first time, whereas *P. gracilis* was isolated previously from the leaf spots of *Achras sapota* (syn. *M. sapota*) [46]. Chemical investigation of the secondary metabolites produced by the fungus led to the isolation of a new azaphilonoid pitholide E (**1**), which possesses 22 carbon atoms and an extra *δ*-lactone ring. In addition, two rare azaphilonoids, pitholide B (**2**) and pitholide D (**3**), were isolated from the genus *Pestalotiopsis* for the first time. Also, this is the first isolation of compounds **4**, **5**, **7** and **8** from *P. microspora*, although they have been previously isolated from *Pestalotiopsis* species. The current study only investigated the fruits of *M. zapota*, so it would be interesting to study endophytic fungal diversity in other parts of *M. zapota* since the secondary metabolites produced by endophytic fungi from this medicinal plant are not well studied.

## Experimental

### General

Extractions were performed using a sonicator (VWR Ultrasound cleaner, model-USC 1700D). Analytical TLC was carried out with silica gel 60 F_254_ precoated aluminum sheets (Merck Art. 1.05554). Compounds on TLC were located using a UV lamp and by heating after spraying with acidic anisaldehyde. Silica gel (Fluka 60741, Merck Art. 7734 and 9385) and Sephadex LH-20 were used for column chromatography. Preparative thin layer chromatography (PTLC) was carried out using silica gel 60 F_254_ precoated glass plates (Merck Art 1.05715). ^1^H NMR and ^13^C NMR were recorded on a Bruker DRX500 (500 MHz for ^1^H and 125 MHz for ^13^C) or JEOL JMN-AL300 (300 MHz for ^1^H and 75 MHz for ^13^C) spectrometer in CD_3_OD or CDCl_3_ solution at 20 °C. Optical rotations were measured on a JASCO P-2200 polarimeter. IR spectra were measured on a JASCO FT/IR-460 spectrometer. UV spectra were recorded on a JASCO MD-4017 photo diode array detector. Positive-ion FABMS were obtained on a JEOL JMX-AX505HA spectrometer.

### Isolation of Endophytic Fungus

Healthy and mature *M. zapota* fruits were purchased from the local market in Kandy, Sri Lanka in March 2014. Fruits were rinsed with running water and triple sterilized with ethanol, 5% NaOCl and distilled water. Interior segments (ca. 10 mm × 5 mm) of sterilized fruits were placed on PDA media in Petri dishes and incubated at room temperature for 5 days. Emerging fungi were serially transferred to PDA media on Petri dishes to obtain pure culture of endophytic fungus.

### Fermentation of Fungus, Extraction, Bioassays and Isolation of Compounds

An endophytic fungus *P. microspora* isolated from the *M. zapota* was cultured by inoculating pure culture grown on potato dextrose agar, in eighty 1 L-conical flasks each containing 400 mL of PDB medium. The flasks were allowed to stand at room temperature for initial 10 days, and shaked every other day on a laboratory shaker (100 rpm). The fermentation medium was filtered after one month and the filtrate was extracted with EtOAc. The residual mycelium was extracted with EtOAc using sonicator. The two EtOAc extracts were combined (23.5 g) since they showed almost identical TLC pattern. The combined EtOAc extract was screened for antioxidant activity using DPPH assay [14], α-amylase inhibitory activity [17], antifungal activity against *Cladosporium cladosporioides* by TLC bioautography method [13], anticandidal activity against five species (*C. albicans, C. parapsilosis, C. glabrata, C. krusei* and *C. tropicalis*) [18], phytotoxicity against *L. sativa* seed germination [16] and brine shrimp toxicity against *Artemia salina* [15]. Chromatographic separation of the combined EtOAc extract was carried out over silica gel (n-hexane–EtOAc-MeOH/n-hexane-CH_2_Cl_2_-MeOH) and Sephadex LH-20 (MeOH), and by PTLC to furnish eight compounds, pitholide E (**1**) (19 mg), together with known pitholide B (**2**) (58 mg), pitholide D (**3**) (26 mg), pestalotin (LL-P880α) (**4**) (6 mg), PC-2 (**5**) (18 mg), LL-P880β (**6**) (28 mg), tyrosol (**7**) (46 mg) and 4-oxo-4*H*-pyran-3-acetic acid (**8**) (24 mg).

Pitholide E (**1**): luminous yellow oil; [α]_D_^25^ − 1080 (*c*, 0.055, MeOH); UV λ_max_ (MeOH): 205, 254, 294, 401 nm; IR ν_max_ (CHCl_3_) 3400, 2958, 2928, 2856, 1750, 1682, 1649, 1550 cm^−1^; ^1^H NMR (CDCl_3_, 500 MHz): δ 7.61 (s, H-4), 7.54 (s, H-1), 5.46 (dd, *J* = 7.0, 3.5 Hz, H-16), 4.53 (s, H-12), 2.29 (s, H_3_-14), 2.10 (m, H-17a), 1.94 (m, H-17b), 1.50 (m, H_2_-18), 1.44 (s, H_3_-15), 1.38–1.25 (m, H_2_-19 to H_2_-22), 0.87 (t, *J* = 7.0 Hz, H_3_-23); ^13^C NMR (CDCl_3_, 125 MHz): δ 168.9 (C-9), 162.62, 162.59, 162.56 (C-3, C-7, C-24), 151.1 (C-1), 138.7 (C-5), 119.9 (C-13), 113.2 (C-8), 108.0 (C-4), 94.3 (C-6), 80.8 (C-11), 79.1 (C-16), 69.9 (C-12), 33.3 (C-17), 31.8 (C-21), 29.3 (C-19), 29.1 (C-20), 24.1 (C-18), 22.6 (C-22), 22.6 (C-15), 19.9 (C-14), 14.1 (C-23), HRFABMS: *m*/*z* 387.1800 [M + H]^+^ (C_22_H_27_O_6_ requires 387.1808).

Pitholide B (**2**): luminous yellow oil; [α]_D_^25^ − 741 (*c*, 0.23, MeOH); ^1^H NMR (CDCl_3_, 500 MHz) *δ* 9.71 (*s*, H-24), 7.99 (*s*, H-4), 7.76 (*s*, H-1), 4.46 (*s*, H-12), 2.96 (*t*, *J* = 7.5 Hz, H-17), 2.36 (H_3_-14), 1.64 (*m*, H_2_-18), 1.40 (*s*, H_3_-15), 1.34–1.20 (*m*, H_2_-19 to H_2_-22), 0.87 (*t*, *J* = 7.0 Hz, H_3_-23); ^13^C NMR (CDCl_3_, 125 MHz) *δ* 198.6 (C-16), 186.8 (C-24), 170.7 (C-9), 166.9 (C-7), 164.2 (C-3), 152.8 (C-1), 143.9 (C-5), 119.5 (C-13), 117.6 (C-8), 107.9 (C-4), 107.4 (C-6), 83.0 (C-11), 68.9 (C-12), 42.4 (C-17), 31.7 (C-21), 29.1 (C-19), 29.1 (C-20), 23.6 (C-18), 22.6 (C-22), 22.4 (C-15), 20.2 (C-14), 14.1 (C-23), ^1^H NMR (CD_3_OD, 500 MHz) *δ* 9.78 (*s*, H-24), 8.02 (*s*, H-4), 7.89 (*d*, *J* = 2.0 Hz, H-1), 4.52 (*s*, H-12), 2.85 (*m*, H_2_-17), 2.37 (*s*, H-14), 1.62 (*m*, H_2_-18), 1.37 (*s*, H_3_-15), 1.37–1.29 (*m*, H_2_-19 to H_2_-22), 0.89 (*t*, *J* = 7.0 Hz, H_3_-23); ^13^C NMR (CD_3_OD, 125 MHz) *δ* 200.7 (C-17), 187.8 (C-24), 172.9 (C-9), 166.6 (C-7), 166.4 (C-3), 154.9 (C-1), 147.2 (C-5), 122.0 (C-13), 119.6 (C-8), 108.4 (C-6), 107.8 (C-4), 84.9 (C-11), 69.7 (C-12), 43.5 (C-17), 30.3 (C-19), 32.9 (C-21), 30.3 (C-20), 24.9 (C-18), 23.7 (C-22), 22.5 (C-17), 20.0 (C-14), 14.4 (C-23); FABMS *m/z*: 387 [M + H]^+^.

Pitholide D (**3**): luminous yellow oil; [α]_D_^25^ − 980 (*c*, 0.077, MeOH); ^1^H NMR (CDCl_3_, 300 MHz) *δ* 7.30 (*s*, H-1), 6.72 (*s*, H-4), 6.10 (*s*, H-6), 4.41 (*s*, H-12), 2.94 (*m*, H_2_-17), 2.18 (*s*, H_3_-14), 1.44 (*s*, H_3_-15), 1.60 (2H, *m*, H-19), 1.39–1.22 (*m*, H_2_-19 to H_2_-22), 0.88 (*t*, *J* = 6.7 Hz, H_3_-23); ^13^C NMR (CDCl_3_, 75 MHz) *δ* 197.6 (C-16), 175.2 (C-7), 161.1 (C-3), 149.0 (C-1), 145.0 (C-5), 119.0 (C-13), 111.9 (C-8), 109.7 (C-4), 102.2 (C-6), 82.5 (C-11), 69.0 (C-12), 41.5 (C-17), 31.7 (C-21), 29.2 (C-19), 29.2 (C-20), 24.3 (C-15), 23.7 (C-18), 22.6 (C-22), 19.6 (C-14), 14.1 (C-23), C-9 signal was ambiguous due to weak signal intensity; FABMS *m/z*: 359 [M + H]^+^.

Pestalotin (LL-P880α) (**4**): white solid; [α]_D_^25^ − 82.3 (*c*, 0.78, MeOH); ^1^H NMR (CDCl_3_, 300 MHz): *δ* 5.15 (*d*, *J* = 1.8 Hz, H-3), 4.30 (*ddd*, *J* = 12.6, 3.9, 3.9 Hz, H-6), 3.76 (*s*, 4-OCH_3_), 3.64 (*m*, H-1′), 2.80 (*ddd*, *J* = 16.8, 12.6, 1.8 Hz, H-5a), 2.25 (*dd*, *J* = 16.8, 3.9 Hz, H-5b), 1.68–1.32 (*m*, H_2_-2′, H_2_-3′, H_2_-4′), 0.92 (*t*, *J* = 7.2 Hz, H_3_-5′); ^13^C NMR (CDCl_3_, 75 MHz): *δ* 173.1 (C-4), 166.7 (C-2), 90.0 (C-3), 78.4 (C-6), 72.5 (C-1′), 56.2 (4-OCH_3_), 32.4 (C-2′), 29.6 (C-5), 27.6 (C-3′), 22.6 (C-4′), 14.0 (C-5′); FABMS *m/z*: 215 [M + H]^+^.

PC-2 (**5**): white solid; [α]_D_^25^ + 76.5 (*c*, 0.62, MeOH); ^1^H NMR (CD_3_OD, 300 MHz) *δ* 6.19 (*d*, *J* = 2.3 Hz, H-5), 5.55 (*d*, *J* = 2.3 Hz, H-3), 4.32 (*dd*, *J* = 7.8, 4.8 Hz, H-1′), 3.86 (*s*, 4-OCH_3_), 1.78 (*m*, H-2′a), 1.65 (*m*, H-2′b), 1.44–1.28 (*m*, H_2_-3′, H_2_-4′), 0.92 (*t*, *J* = 7.2 Hz, H_3_-5′); ^13^C NMR (CD_3_OD, 75 MHz): *δ* 173.8 (C-4), 169.0 (C-6), 167.1 (C-2), 99.8 (C-5), 88.6 (C-3), 71.1 (C-1′), 57.0 (4-OCH_3_), 35.8 (C-2′), 28.4 (C-3′), 23.5 (C-4′), 14.3 (C-5′); FABMS *m/z*: 213 [M + H]^+^.

LL-P880β (**6**): white solid; [α]_D_^25^ − 61.2 (*c*, 0.23, MeOH); ^1^H NMR (CDCl_3_, 500 MHz) *δ* 5.14 (*d*, *J* = 1.7 Hz, H-3), 4.51 (*ddd*, *J* = 12.8, 3.9, 3.9 Hz, H-6), 3.80 (*m*, H-2′), 3.76 (*s*, 4-OCH_3_), 3.49 (*m*, H-1′), 2.89 (*m*, H-5a), 2.32 (*dd*, *J* = 17.2, 3.9 Hz, H-5b), 1.63 (*m*, H-3′a), 1.52 (*m*, H-3′b), 1.52 (*m*, H-4′a), 1.40 (*m*, H-4′b), 0.95 (*t*, *J* = 7.2 Hz, 5′-H_3_); ^13^C NMR (CDCl_3_, 75 MHz) *δ* 173.3 (C-4), 166.3 (C-2), 89.8 (C-3), 78.0 (C-6), 73.8 (C-1′), 71.0 (C-2′), 56.2 (4-OCH_3_), 36.0 (C-3′), 29.4 (C-5), 18.8 (C-4′), 13.9 (C-5′); FABMS *m/z*: 231 [M + H]^+^.

Tyrosol (**7**): white solid; ^1^H NMR (CDCl_3_, 300 MHz) *δ* 7.10 (*d*, *J* = 8.4 Hz, H-3, H-5), 6.79 (*d*, *J* = 8.4 Hz, H-2, H-6), 3.83 (*t*, *J* = 6.6 Hz, H_2_-2′), 2.81 (*t*, *J* = 6.6 Hz, H_2_-1′); ^13^C NMR (CDCl_3_, 75 MHz) *δ* 154.3 (C-4), 115.4 (C-3, C-5), 130.1 (C-2, C-6), 130.3 (C-1′), 63.8 (C-2′), 38.2 (C-1′); FABMS *m/z*: 139 [M + H]^+^.

4-Oxo-4*H*-pyran-3-acetic acid (**8**): white solid; ^1^H NMR (CD_3_OD, 500 MHz) *δ* 8.11 (*d*, *J* = 1.0 Hz, H-2), 8.04 (*dd*, *J* = 7.0, 1.0 Hz, H-6), 6.39 (*d*, *J* = 7.0 Hz, H-5), 3.52 (*s*, H_2_-7); ^13^C NMR (CD_3_OD, 125 MHz,) *δ* 180.2 (C-4), 174.0 (C-8, signal was not clearly observed but assigned on the basis of the HMBC correlation), 158.6 (C-2), 156.9 (C-6), 125.8 (C-3), 117.1 (C-5), 31.6 (C-7); ^1^HNMR (CDCl_3_, 300 MHz) *δ* 7.92 (*s*, H-2), 7.87 (*d*, *J* = 5.4 Hz, H-6), 6.51 (*d*, *J* = 5.4 Hz, H-5), 3.47 (*s*, H_2_-7); ^13^CNMR (CDCl_3_, 75 MHz,) *δ* 179.5 (C-4), 156.4 (C-2), 154.5 (C-6), 123.8 (C-3), 116.7 (C-5), 29.7 (C-7); ^1^H NMR (D_2_O, 300 MHz, referenced to internal dioxane at δ 3.53) *δ* 7.96 (*s*, H-2), 7.93 (*d*, *J* = 5.7 Hz, H-6), 6.32 (*d*, *J* = 5.7 Hz, H-5), 3.23 (*s*, H_2_-7); ^13^CNMR (D_2_O, 75 MHz, referenced to internal dioxane at δ 67.4) *δ* 181.8 (C-4), 176.2 (C-8), 159.4 (C-2), 157.7 (C-6), 124.5 (C-3), 116.5 (C-5), 32.1 (C-7); FABMS *m/z*: 155 [M + H]^+^.
